# Genetic Effects of *FTO* and *MC4R* Polymorphisms on Body Mass in Constitutional Types

**DOI:** 10.1093/ecam/nep162

**Published:** 2011-02-13

**Authors:** Seongwon Cha, Imhoi Koo, Byung L. Park, Sangkyun Jeong, Sun M. Choi, Kil S. Kim, Hyoung D. Shin, Jong Y. Kim

**Affiliations:** ^1^Division of Constitutional Medicine Research, Korea Institute of Oriental Medicine, 483 Exporo, Yuseong-gu, Daejeon, 305-811, Republic of Korea; ^2^Department of Genetic Epidemiology, SNP Genetics Inc., Geumcheon-gu, Seoul, Republic of Korea; ^3^Kirin Oriental Medical Hospital, Seoul, Republic of Korea; ^4^Department of Life Science, Sogang University, Mapo-gu, Seoul, Republic of Korea

## Abstract

Sasang constitutional medicine (SCM), a Korean tailored medicine, categorizes human beings into four types through states of physiological imbalances and responsiveness to herbal medicine. One SCM type susceptible to obesity seems sensitive to energy intake due to an imbalance toward preserving energy. Common variants of fat mass and obesity associated (*FTO*) and melanocortin 4 receptor (*MC4R*) genes have been associated with increased body mass index (BMI) by affecting energy intake. Here, we statistically examined the association of *FTO* and *MC4R* polymorphisms with BMI in two populations with 1370 Koreans before and after SCM typing, and with the lowering of BMI in 538 individuals who underwent a 1-month lifestyle intervention. The increased BMI replicated the association with *FTO* haplotypes (effect size *≃* 1.1 kg/m^2^) and *MC4R* variants (effect size *≃* 0.64 kg/m^2^). After the lifestyle intervention, the carriers of the haplotype represented by the minor allele of rs1075440 had a tendency to lose more waist-to-hip ratio (0.76%) than non-carriers. The constitutional discrepancy for the accumulation of body mass by the effects of *FTO* and/or *MC4R* variants seemed to reflect the physique differences shown in each group of SCM constitutional types. In conclusion, *FTO* and *MC4R* polymorphisms appear to play an important role in weight gain, while only *FTO* variants play a role in weight loss after lifestyle intervention. Different trends were observed among individuals of SCM types, especially for weight gain. Therefore, classification of individuals based on physiological imbalance would offer a good genetic stratification system in assessing the effects of obesity genes.

## 1. Introduction

Common variations in the first intron of the fat mass and obesity associated (*FTO*) gene, have been shown to be risk factors for obesity in several studies of Caucasian [1–3], Asian [4–9] and African American [10] subjects. However, conflicting results have been generated in certain Asian (e.g., Japanese and Chinese) [11, 12] and African American [3] populations. These inconsistencies may stem from certain genetic or non-genetic factors that were not taken into account by the study authors [13].

For these reasons, we became interested in a traditional Korean medicine approach that uses constitutional typing and novel stratification criteria to assess the risk factors for obesity in humans with different genetic compositions. Sasang constitutional medicine (SCM), a branch of traditional Korean medicine, is a unique personalized medical system that classifies humans into four constitutional types based primarily on their responses to herbal medicine, along with an individual's physiological, psychological and physical characteristics. Recently, there have been efforts to explain the constitution concept of SCM with the language of modern genetics [14], and to search for a genetic linkage to SCM on a genome-wide scale [15]. The underlying principle of this classification stems from the concept that each individual is born in one of four physiologically unbalanced states: the Taeyang (TY), Tae-eum (TE), Soyang (SY) or Soeum (SE) [16, 17]. The TE type is dominant in preservative function (mainly by liver and small intestine) over respiratory function (mainly by lung), whereas the TY type has an opposite dominance. This interrelation is also maintained between the SY and SE types, that is, the SY type has strong digestive function (mainly by stomach and pancreas) over excretive function (mainly by kidney and large intestine), whereas the SE type has a countertrend. Therefore, the TE type is thought to display a strong physiological tendency to become obese by preserving energy; TE types tend to gain more weight and achieve a larger waist circumference than other types [17]. Bioelectrical impedance analyses have shown that the TE type is associated with a high body fat mass [18]. Thus, it may be possible to achieve more delicate and stringent analyses of the risk factors for obesity via the stratification of populations according to SCM typing.

The *FTO* gene has been associated with food intake [19–21] and reduced satiety response [22], and the genetic effect on obesity is attenuated by physical activity [23]. However, this gene does not appear to influence changes in body weight or fat distribution in people who have undergone 1 or 2 years of intervention (e.g., exercise and dietary control) [24, 25]. We are also interested in the melanocortin 4 receptor (*MC4R*) gene. *MC4R* is similar to *FTO* in terms of its role in the regulation of food intake, although it has an additional effect on energy expenditure [26]. Recent two genome-wide association studies revealed that common allelic variants in *MC4R* locus are associated with obesity-related phenotypes [27, 28]. In addition, the common variants near *MC4R* have been found to have additive effects on body mass index (BMI) along with *FTO* variants [28].

Here, we examine the genetic effects of *FTO* and *MC4R* variants on obesity-related phenotypes. We assessed the relationship between lifestyle intervention and common variants of the genes with obesity-related phenotypes in individuals of each SCM type.

## 2. Methods

### 2.1. Subjects

We recruited 835 individuals from an obesity clinic in Kirin Oriental Medical Hospital (Seoul, Korea) (this population is hereafter named Kirin) between 2001 and 2004, and 535 individuals were recruited from 12 other oriental medical hospitals in Korea (this population is hereafter named Multicenter) during a 2-year period starting from 2007. The enrolled subjects did not include patients with chronic diseases, such as hypertension, coronary artery disease, stroke, diabetes, hyperlipidemia, and so forth. Written informed consent was obtained from all subjects and the Institutional Review Board of the Korea Institute of Oriental Medicine approved the study protocol. Abdominal fat area was measured using computerized tomography (CT) cross-sectional images, as previously described [29] (Hispeed CT/e, GE, USA). Blood samples were obtained for biochemical measurements, and DNA was extracted from each subject and stored at −70°C until further use. The characteristics of the recruited individuals are shown in Table 1. There were more women than men in our study population (Table 1), possibly because women tend to attend obesity clinic more frequently than men do. Therefore, we considered sex as a covariate in our linear regression analyses to minimize the effects of this bias.

### 2.2. Subgrouping by Sasang Constitution

The classification of Sasang constitution is performed based on three main factors: physique and facial appearance, psychological character, and physiological and pathological reactions. As for the appearance, individuals with TE type tend to have bigger physiques than those with SY and SE types [18], and the subjects of SY type usually have smaller hip than those of SE type. Individuals with SE and SY types have psychological characters reciprocal to each other. The SY type subjects, more than the SE types, have been found to be more extroverted in their performance on Myers-Briggs Type Indicator [18] and to show higher score for Novelty Seeking and lower score for Harm Avoidance from Temperament and Character Inventory [30]. Because each type has been known to have type-specific physiological imbalances as described in the introduction [16, 17], important pathological symptoms are different among the Sasang types: bowel movements and constipation for the SY type, perspiration for the TE type and digestion for the SE type.

The constitutional types of the 835 subjects recruited from the obesity clinic were categorized by an oriental medical doctor with the self-reported Questionnaire for the Sasang Constitution Classification (QSCC) [18] using Win QSCC II software, version 99 (Ssord Medicom & Ssord OMS, Seoul, Korea). QSCC including the questionnaires on the three main factors for constitution classification was developed in 1993 and upgraded to the present version (QSCC II) in 1996 [31]. In the latter version, 1366 individuals (48.9% males; age 10–60 years) whose constitutions had been determined by professional clinicians were used for standardization and 265 subjects were assessed for validation. The internal consistency of each constitution was measured by Cronbach's *α*: 0.57 for TY type, 0.59 for TE type, 0.57 for SY type and 0.63 for SE type. The diagnostic discriminability of QSCC II was 70% overall in hit-ratio (75% for TE type, 60% for SY type, 71% for SE type). Professional SCM doctors, taking into account the typing from the questionnaire, categorized the constitutional types of the 535 subjects according to responsiveness to herbal medicine and diagnosis. The constitutional types of 24 subjects in the Multicenter population were not characterized.

### 2.3. Weight-Control Program

After removing subjects who had not fulfilled a 1-month weight-control program, a subgroup of subjects (*n=538*) from the Kirin population was selected to be analyzed for the genetic effects of *FTO* and *MC4R* variants on weight loss after the 1-month lifestyle intervention. The intervention program consisted of a low-calorie diet (*∼*600 kcal per day: breakfast with uncooked food 85 kcal and soybean milk 120 kcal, lunch 200 kcal, Chegamuiyiin-tang 186.3 kcal), daily exercise using a treadmill machine (5 km/h for 45 min to 1 hr), an electrolipolysis treatment using Lipodren equipment (Sormedic, Barcelona, Spain) and the administration of Chegamuiyiin-tang (three times per day) containing 17 herbs. The prescription of Chegamuiyiin-tang for 1 day was as follows: Cocicis Semen 66 g, Rehmanniae Radix Preparata 33 g, Angelicae Gigantis Radix 16 g, Raphani Semen 12 g, Akebiae Lignum 12 g, Plantaginis Semen 12 g, Astragali Radix 12 g, Gastrodiae Rhizoma 12 g, Mori Cotex Radicis 12 g, Glycyrrhizae Radix 12 g, Thujae Semen 12 g, Lycii Fructus 8 g, Cnidii Rhizoma 4 g, Carthami Flos 4 g, Caesalpiniae Lignum 4 g, Cervi Parvum Cornu 4 g and Cervi Cornu 12 g. The subjects were asked to keep a daily diet diary to help maintain the low-calorie diet. Changes in anthropometric features were measured by a bio-impedance analysis using a commercial device (Inbody 2.0 Biospace, Seoul, Korea).

### 2.4. Genotyping of Single Nucleotide Polymorphism

Genotyping was performed using the TaqMan system [32] with amplifying primers and TaqMan probes (Applied Biosystems, Foster City, CA, USA). Two FAM and VIC dyes were labeled on each allelic probe. PCR was performed in 384-well plates. Genomic templates were amplified in a total reaction volume of 5 *μ*l, containing TaqMan Universal Master Mix (Applied Biosystems), 900 nmol/l of PCR primer, 200 mmol/l of TaqMan probe and 20 ng of genomic DNA. Amplification was performed via a heating cycle (50°C for 2 min and 95°C for 10 min), followed by an amplifying cycle (40 cycles of 95°C for 15 s, 60°C for 1 min) in a thermal cycler (PE 9700, Applied Biosystems). Fluorescence intensity was measured using a Prism 7900HT (Applied Biosystems).

### 2.5. Statistics

We used the *χ* ^2^-test to determine if the variants of *FTO* and *MC4R* were in Hardy-Weinberg equilibrium. Analysis of variance was used to check if the characteristics in Table 1 differed according to SCM types. Linkage disequilibrium (LD; Lewontin's *D*′ = *D*/|*D*_max _|) and haplotype structures were obtained from the Haploview program, version 4.1 (Daly Lab at the Broad Institute, Cambridge, MA, USA) [33]. Haplotypes of the *FTO* single nucleotide polymorphism (SNPs) were constructed using Phase (version 2.1), a Bayesian algorithm-based program [34, 35]. The relationships of *FTO* and *MC4R* with obesity-related traits were analyzed via linear regression analyses, with adjustments for age and gender as covariates. Meta-analysis was performed in a mixed model using SAS, according to the instruction previously reported [36]. We use Storey's q-value method of measuring for the minimum false discovery rate to control the significant level *P*-value for each hypothesis test related to haplotype or SNP in multiple comparisons [37]. Effect sizes were presented as changes in the minor allele carriage, determined via a linear regression analysis. All statistical analyses were performed using SAS, version 8.02 (SAS, Cary, NC, USA) and Matlab, version 7.6 (MathWorks, Natick, MA, USA).

## 3. Results

### 3.1. Characteristics of Common Variants

**Figure 1:**
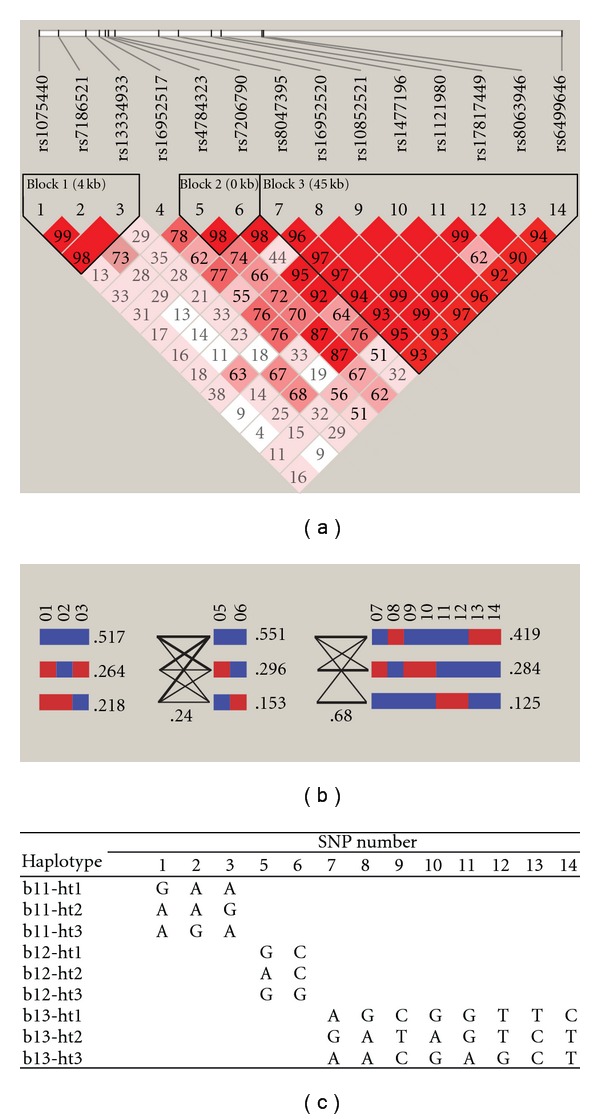
The LD (a) and haplotype (b) structures of *FTO* were determined via Haploview program. In *FTO* haplotypes, blue and red colors indicate major and minor alleles of each SNP, while thick and thin lines between blocks indicate >10% and >1% connecting frequencies, respectively, between haplotypes in neighboring blocks (b). (c) Among the 14 SNPs previously reported to reside in the first intron of *FTO*, three, two and eight SNPs are located in block 1 (bl1), block 2 (bl2) and block 3 (bl3), respectively.

Using international Japanese and Chinese HapMap databases, we searched 23 validated SNPs that were previously reported to reside in the first intron of *FTO* [1, 2] (Supplementary Table 1). The Chinese and Japanese HapMap databases provide a robust platform for studies in a Korean population. We then constructed the LD structure using 14 SNPs (Figure 1(a)) that did not demonstrate complete LD with other SNPs and did not have minor allele frequencies <10%. This resulted in three LD blocks. Each block consisted of three haplotypes, which were selected using a cutoff of 10% haplotype frequencies (Figures 1(b) and 1(c)). Haplotypes were named with the abbreviated LD block name, followed by the haplotypes (in order of frequency) in each block (e.g., bl3-ht1 indicates the most frequent haplotype in LD block 3). In the cases of bl1-ht1 and bl2-ht1 with over 50% haplotype frequencies (Figure 1(b)), we used the other minor haplotypes in each block as interesting risk haplotypes in order to avoid confusion in interpreting the association results and designated them bl1-ht1r and bl2-ht1r (“r” indicates risk). We selected two common variants (rs17782313 and rs12970134) near *MC4R* that had previously been reported in two independent genome-wide association studies [27, 28]. Two haplotypes of *FTO*, bl2-ht3 and bl3-ht3, were more frequent (3-4%) in the Kirin population than in the Multicenter population, as the individuals in Kirin were heavier. Similarly, two SNPs near *MC4R* were more frequent (3-4%) in the Kirin population. The *FTO* and *MC4R* alleles were determined to be in Hardy-Weinberg equilibrium in each population (*P>.05*).

### 3.2. Increased Effects of FTO and MC4R in the TE Type

Association analyses were performed on the three haplotypes in each LD block of *FTO* and two SNPs of *MC4R* in two Korean populations. We observed that haplotype frequencies of *FTO* were correlated with SCM type in a Multicenter population, but that minor allele frequencies of *MC4R* SNPs were not related with SCM type in the Multicenter population. The effect size (i.e. the unit change caused by the carriage of risk allele) was calculated via linear regression analysis under an additive model as done in previous reports.

**Figure 2:**
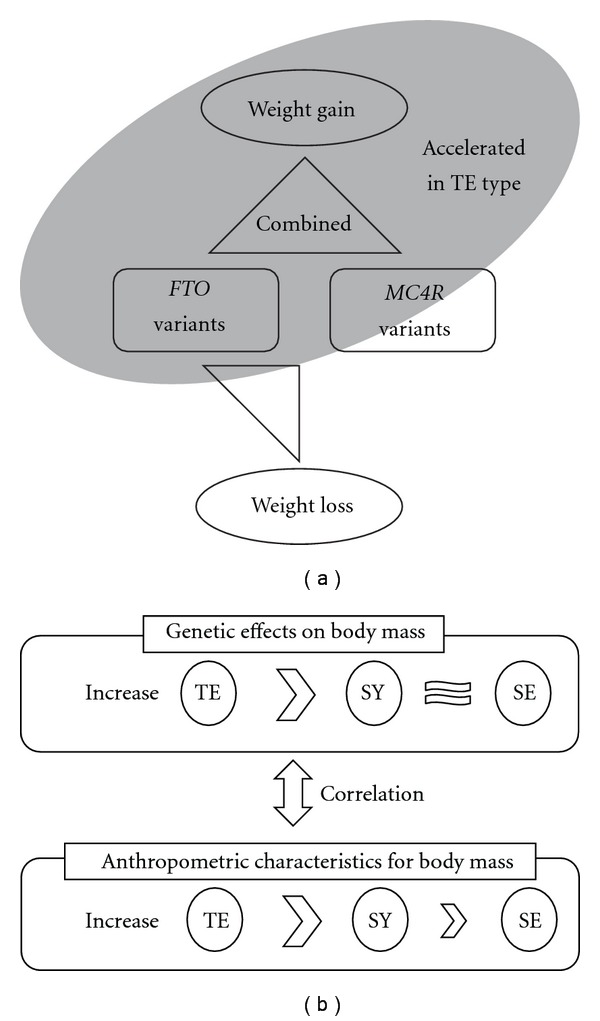
A hypothetical diagram on the relationships between the variants of *FTO* and/or *MC4R* and weight changes in the context of TE constitutional type (a). The interaction of the obesity-related variants and TE type may be strong for weight gain especially in a heavy population (Kirin population in this study). Strong correlation was found between the effects of *FTO* haplotypes and *MC4R* SNPs on body mass (BMI and WHR) and clinically measured body mass (BMI and WHR) in each SCM type (b). The action of the genes in the context of SCM type may differently affect body mass changes in each type. The size of inequality sign indicates the size of body mass difference between SCM types.

For the Kirin population, two *FTO* haplotypes (bl2-ht3 and bl3-ht3) were associated with increased BMI in all subjects and the subjects of TE types (Supplementary Table 2). Both bl2-ht3 and bl3-ht3 showed stronger effect sizes in TE types (for both bl2-ht3 and bl3-ht3, there was a 1.4-fold increase in effect size) than in all subjects (Figure 2(a)). The minor alleles of three *FTO* SNPs tagging the above haplotypes (rs7206790 in bl2-ht3, rs17817449 and rs1121980 in bl3-ht3) were also associated with increased BMI in TE types (Supplementary Table 3). Two *MC4R* SNPs were also associated with increased BMI (Supplementary Table 2) in all subjects and the subjects of TE types, as previously reported [27, 28]. There were no genetic effects of *FTO* and *MC4R* in SY and SE types. For the Multicenter population, the statistical analyses performed using a dominant model because the number of minor allele homozygotes of three SNPs (rs7206790, rs1121980 and rs17817449) was very small. We did not find any associations of *FTO* or *MC4R* in the Multicenter population (Supplementary Table 4).

In combining the two populations, we can find the strong association of *FTO* haplotypes (bl2-ht1r, bl2-ht3 and bl3-ht3) and *MC4R* SNPs (rs17782313 and rs12970134) with increased BMI in all subjects (Supplementary Table 5). In SCM types, two *FTO* haplotypes (bl2-ht3 and bl3-ht3) had an effect on BMI increase only in subjects of TE type. When we searched the combinatorial effects of *FTO* and *MC4R* variants, we found that the association signal of *FTO* haplotypes on BMI became stronger (for bl2-ht3, 2.0-fold increase of effect size in Kirin population and 1.5-fold increase in combined population; for bl3-ht3, 2.1-fold increase of effect size in Kirin population and 1.7-fold increase in combined population) in the minor allele carriers of *MC4R* rs17782313 (Supplementary Table 6).

### 3.3. Weight Loss in Response to Lifestyle Intervention

A subgroup of subjects (*n=538*) recruited from a Kirin population participated in a 1-month weight-control program to assess the relationship between the obesity-related genes and body fat mass in response to lifestyle intervention. The percentage of body mass change is shown in Table 1. One percent indicates one-hundredth of the value of body mass change after the weight-control program divided by the value before the program. Obesity-related phenotypes including BMI and waist-to-hip ratio (WHR) were reduced after participation in the weight-control program. On average, the BMI and WHR decreased by 7.9% and 3.1%, respectively. Interestingly, the subjects of SY types showed only a small decrease of BMI (5.7%) and WHR (1.9%), compared with those of other types (*P=.00054* for BMI loss and *P=.00017* for WHR loss).

Statistical analyses were performed using a dominant model because the number of minor allele homozygotes was very small after stratifying by SCM type. We found an association of an *FTO* haplotype (bl1-ht1r) with WHR loss in all subjects (Supplementary Table 7), but not with BMI loss in all subjects (Supplementary Table 8). When we stratified the subjects according to SCM type, the *FTO* haplotypes appeared to be related with BMI loss only in SY type subjects (Supplementary Table 8). However, we cannot conclude that there is an association between *FTO* haplotypes and BMI loss in the subjects of SY type, because the number of SY type subjects is very small. A replication analysis with another population is necessary to confirm the relationship between the haplotype and BMI loss in the subjects of SY type. We did not find any relationships between *MC4R* and the percentage of body mass change.

## 4. Discussion

Consistent with previous studies, we replicated the association of *MC4R* with BMI in a Korean population for the first time and we also confirmed the association of the *FTO* LD block with obesity-related phenotypes in Koreans. Our findings revealed that the haplotypes represented by minor alleles of three SNPs (rs17817449, rs1121980 and rs7206790) located within the first intron of *FTO* were associated with body mass (Supplementary Tables 2 and 5). The association of two SNPs near *MC4R* with BMI was well replicated in the Kirin population (26.5 kg/m^2^), therefore the association of *MC4R* variants with BMI could be found in the heavy population. To our knowledge, this replication with the common variants near *MC4R* is the first report in an East Asian population, although there have been several replicated results in Caucasians [27, 28]. As previously reported [28], the variants of *FTO* and *MC4R* had an additive effect on BMI increase, because the risk allele carriers of both *FTO* and *MC4R* variants showed approximately 2.0-fold strengthened susceptibility to body mass accumulation.

We expected that the effects of *FTO* and *MC4R* on body fat mass would be more apparent if the population were stratified by SCM type, as a previous report [18] has demonstrated distinct susceptibilities to obesity among subjects with different SCM types. Our recruited populations consisted of a large proportion of subjects with the TE type (Table 1). This was not surprising, considering that the TE type belongs to almost half of the Koreans and is prevalent in overweight populations [18, 38]. However, it would have been optimal to recruit additional subjects of the SY and SE types to boost the statistical power of our analysis.

When the population was stratified by SCM type (excluding subjects with the rare TY type; 0.6% of our study population), we observed different relationships of *FTO* and *MC4R* with obesity-related phenotypes (Supplementary Tables 2 and 5). In meta-analyses, *MC4R* SNPs had no significant effect on BMI increase in the subjects of TE type; therefore *MC4R* variants might show a strong association with BMI in the heavier Kirin population (mean BMI of TE type: 27.2 kg/m^2^) than in the Multicenter population (mean BMI of TE type: 25.0 kg/m^2^) (Figure 2(a)). The relationship between obesity and the TE type by the risk alleles of *FTO* and *MC4R* was consistent with the findings of a previous report, which indicated that TE subjects tend to have higher fat masses [18]. In the subjects of SE and SY types, we observed no relationship between variants and body mass. Therefore, specific alleles may confer different susceptibilities to increased body mass in subjects with TE type or not; the genetic differences appear to be similar to the clinical differences in BMI between TE type and the other types (Table 1 and Figure 2).

The genetic factors in the same haplotype may exert different effects on *FTO* expression in TE type subjects over other type subjects, in response to energy-preserving action including increased nutritional intake, decreased physical activity and so on. The interaction of the haplotypes and SCM types might be important for weight gain (Figure 2(a)). Therefore, the values of body mass in the subjects of SCM TE type and other types (Table 1) also corresponded with the effects of *FTO* haplotypes in the subjects of TE type and others (Figure 2(b)).

We revealed that individuals harboring the high-risk allele of *FTO* (also with *MC4R*) had no more significant propensity for BMI loss than other individuals. This corresponded with the information from previous studies that *FTO* does not appear to influence changes in body mass with the lifestyle intervention using exercise and dietary control [24, 25]. However, WHR loss was significantly associated with *FTO* haplotype (bl1-ht1r) in all subjects (Supplementary Table 7). Two previous reports on lifestyle interventions have shown an association with rs8050136 and rs9939609. Both SNPs are in complete LD with rs17817449 in a white population [1] and rs9939609 is in complete LD with rs17817449 in the population evaluated here. We similarly did not find any effect of loss in body mass with rs17817449 (Supplementary Table 9). Therefore, it would be necessary to search the effect on weight loss with another *FTO* variant (e.g., rs1075440 constructing bl1-ht1r haplotype).

The *FTO* is abundantly expressed in the hypothalamic nucleus, where it helps regulate energy homeostasis [2, 39]. A previous study in mice revealed that *FTO* mRNA expression (*FTO* encodes a 2-oxoglutarate-dependent nucleic acid demethylase) is regulated by nutritional intake and that it oscillates in response to feeding and fasting [39]. After a 1-month weight control containing low-calorie diet, the carriers of the obesity-risk alleles might undergo significant changes in *FTO* mRNA expression within the hypothalamic nuclei. Thus, future studies should examine the genetic factors that regulate *FTO* expression and attempt to link the methylation status of *FTO* to changes in body mass.

Consistent with previous reports [2, 27], accumulation in WHR by the action of *FTO* and *MC4R* variants was observed in all subjects and TE type subjects (Supplementary Table 10). Increased abdominal fat mass was associated with the variants of *FTO* and *MC4R* only in TE type subjects (Supplementary Table 11). This phenomenon is consistent with the tendency of TE type subjects to have thicker waistlines [17]. Therefore, we can see constitutional difference in the case of waist mass accumulation between TE type and the other types (Figure 2).

In conclusion, our findings demonstrate that the association of *FTO* and/or *MC4R* with increased weight gain in all subjects was primarily maintained in the subjects of TE type, who are considered to have a tendency to preserve energy rather than to expend it [17]. The genetic background of subjects differs according to SCM type (especially TE type), much like genetic background differs in subjects of different ethnicities and sex. Ethnic differences may be caused by the compositions of each SCM type in the populations, thereby accounting for the controversial results observed in several previous Asian studies. Specifically, when certain ethnic populations have fewer members with TE type and more members with SY and SE types, the relationship between the *FTO* gene and obesity may become weak. It would therefore be helpful to stratify the people according to physiological constitution types, as seen in SCM, to precisely assess the genetic effects on obesity-related phenotypes.

## Supplementary Data

Supplementary data are available at *eCAM* online.

## Funding

Korea Science and Engineering Foundation (KOSEF) grant funded by the Korean government (MEST) (Grant No. M10643020004-08N4302-00400).

## Supplementary Material

Figure S1: (a) Meta-analysis plot (adult cohorts) showing the rs9939609 per-A allele effect size on BMI, expressed in log10BMI Z-score units. (b) Meta-analysis plot (adult cohorts) showing the rs9939609 per-A allele effect size on BMI in males only, expressed in log10BMI Zscore units. (c) Meta-analysis plot (adult cohorts) showing the rs9939609 per-A allele effect size on BMI in females only, expressed in log10BMI Zscore units.
Figure S2: (a) Association, gene structure, conservation, linkage disequilibrium and recombination for the FTO gene region. (a) T2D association in initial WTCCC study. A. Plot of - log(p-values) (Yaxis) for T2D against chromosome position in Mb(X-axis); B. Genomic location of genes showing intron and exon structure (NCBI BUILD 35); C. Multiz vertebrate alignment of 17 species showing evolutionary conservation; D. GOLDsurfer plot of linkage disequilibrium in CCC cases. Values given as pairwise r2; E. Recombination rate given as cM/MB. Red lines represent recombination hotspots (HapMap); F. GOLDsurfer plot of linkage disequilibrium in HapMap CEU samples, values given as pairwise r2. (b) BMI association in initial WTCCC study. A. Plot of -log(p-values) (Y-axis) for T2D against chromosome position in Mb(X-axis); B. Genomic location of genesshowing intron and exon structure (NCBI BUILD 35); C. Multiz vertebrate alignment of 17 species showing evolutionary conservation; D. GOLDsurfer plot of linkage disequilibrium in CCC cases. Values given as pairwise r2; E. Recombination rate given as cM/MB. Red lines represent recombination hotspots (HapMap); F. GOLDsurfer plot of linkage disequilibrium in HapMap CEU samples, values given as pairwise r2.
Figure S3: (a) Expression profile of the FTO gene. The relative expression level of the FTO gene is given for a range of human tissues. Figures on the Y-axis refer to the abundance of FTO mRNA relative to B2M and BGUS and are normalised to adult human pancreas. (b) Expression profile of KIAA1005. The relative expression level of the KIAA1005 gene is given for a range of human tissues. Figures on the Y-axis refer to the abundance of KIAA1005 mRNA relative to B2M and BGUS and are normalised to adult human pancreas.
Table S1. Basic clinical characteristics of all studies.
Table S2(a). Genotype counts in normal weight and overweight (BMI ≥25kg/m2) individuals from all studies – children and adults. Odds ratios and P values are corrected for sex. (b). Genotype counts in normal weight and obese (BMI ≥30kg/m2) individuals from all studies – children and adults. Odds ratios and P values are corrected for sex.
Table S3(a). Ponderal Index, corrected for sex and gestation in individuals born at 36.00 weeks' gestation or later (twins excluded). (b) The association of weight with rs9939609 genotypes, corrected for sex in a) type 2 diabetes cases from genomewide and replication studies; b) control participants from genome-wide and replication studies and c) adult population studies. P values represent per-A allele effects. (c). Children: weight and height corrected for sex. (d) The association of height with rs9939609 genotypes, corrected for sex in a) type 2 diabetes cases from genome-wide and replication studies; b) control participants from genome-wide and replication studies and c) adult population studies. P values represent per-A allele effects. (e) The association of waist circumference with rs9939609 genotypes, corrected for sex in a) type 2 diabetes cases from genome-wide and replication studies; b) control participants from genome-wide and replication studies and c) adult population studies. P values represent per-A allele effects. (f) The association of various measures of subcutaneous fat with rs9939609 genotypes, corrected for sex in four studies. P values represent per-A allele effects.
Table S4. Sequencing variants of FTO gene, observed in 47 individuals with BMI >40 kg/m2.

## Figures and Tables

**Table 1:** Anthropometric and clinical characteristics of the subjects^a^.

Feature	All	TE	SY	SE	*P*-value*
Kirin population					
Number (*n*)	835	685	81	69	—
Female (%)	93.4	92.1	98.8	100	—
Age (years)	27.7 ± 8.09	27.7 ± 8.01	28.8 ± 9.54	27.0 ± 6.93	.39
BMI (kg/m^2^)	26.5 ± 4.66	27.2 ± 4.63	23.6 ± 3.48	23.0 ± 3.19	**8.1 × 10^-**20**^**
WHR	0.888 ± 0.0755	0.897 ± 0.0759	0.849 ± 0.0578	0.840 ± 0.0549	**2.4 × 10^-**14**^**
BMI loss (%)^a^	−7.86 ± 4.10 (538)	−8.12 ± 3.98 (443)	−5.74 ± 4.55 (48)	−7.55 ± 4.16 (47)	**5.4 × 10^-**4**^**
WHR loss (%)^a^	−3.12 ± 2.52 (538)	−3.32 ± 2.45 (443)	−1.87 ± 2.46 (48)	−2.54 ± 2.82 (47)	**1.7 × 10^-**4**^**
Multicenter population					
Number (*n*)	535	196	172	137	—
Female (%)	64.2	57.1	70.9	66.9	—
Age (years)	42.6 ± 13.1	43.7 ± 13.9	43.0 ± 12.1	41.1 ± 12.9	.18
BMI (kg/m^2^)	22.9 ± 3.09	25.0 ± 2.95	22.4 ± 2.54	20.9 ± 2.01	**2.8 × 10^-**30**^**
WHR	0.885 ± 0.0736	0.915 ± 0.0632	0.876 ± 0.0671	0.858 ± 0.0806	**5.0 × 10^-**11**^**

The values are given as mean ± SD; ^a^The percentage of a loss in body mass was calculated as one-hundredth of the value of body mass change after the weight-control program divided by the value before the program. The number of subjects participated in the program are indicated in parentheses; *The *P*-value (*P* < .05, boldface) was calculated with analysis of variance.
