# Supplementary material for: Genetic Effects of FTO and MC4R Polymorphisms on Body Mass in Constitutional Types
Source: Evid Based Complement Alternat Med. 2011 Feb 13;2011:106390. doi: 10.1093/ecam/nep162 (PMC3094695; doi:10.1093/ecam/nep162)
Supplement: Supplementary file 1 — Figure S1: (a) Meta-analysis plot (adult cohorts) showing the rs9939609 per-A allele effect size on BMI, expressed in log10BMI Z-score units. (b) Meta-analysis plot (adult cohorts) showing the rs9939609 per-A allele effect size on BMI in males only, expressed in log10BMI Zscore units. (c) Meta-analysis plot (adult cohorts) showing the rs9939609 per-A allele effect size on BMI in females only, expressed in log10BMI Zscore units. Figure S2: (a) Association, gene structure, conservation, linkage disequilibrium and recombination for the FTO gene region. (a) T2D association in initial WTCCC study. A. Plot of - log(p-values) (Yaxis) for T2D against chromosome position in Mb(X-axis); B. Genomic location of genes showing intron and exon structure (NCBI BUILD 35); C. Multiz vertebrate alignment of 17 species showing evolutionary conservation; D. GOLDsurfer plot of linkage disequilibrium in CCC cases. Values given as pairwise r2; E. Recombination rate given as cM/MB. Red lines represent recombination hotspots (HapMap); F. GOLDsurfer plot of linkage disequilibrium in HapMap CEU samples, values given as pairwise r2. (b) BMI association in initial WTCCC study. A. Plot of -log(p-values) (Y-axis) for T2D against chromosome position in Mb(X-axis); B. Genomic location of genesshowing intron and exon structure (NCBI BUILD 35); C. Multiz vertebrate alignment of 17 species showing evolutionary conservation; D. GOLDsurfer plot of linkage disequilibrium in CCC cases. Values given as pairwise r2; E. Recombination rate given as cM/MB. Red lines represent recombination hotspots (HapMap); F. GOLDsurfer plot of linkage disequilibrium in HapMap CEU samples, values given as pairwise r2. Figure S3: (a) Expression profile of the FTO gene. The relative expression level of the FTO gene is given for a range of human tissues. Figures on the Y-axis refer to the abundance of FTO mRNA relative to B2M and BGUS and are normalised to adult human pancreas. (b) Expression profile of KIAA1005. The relative ex [file 106390.f1.pdf]

Supporting figures
Figure S1

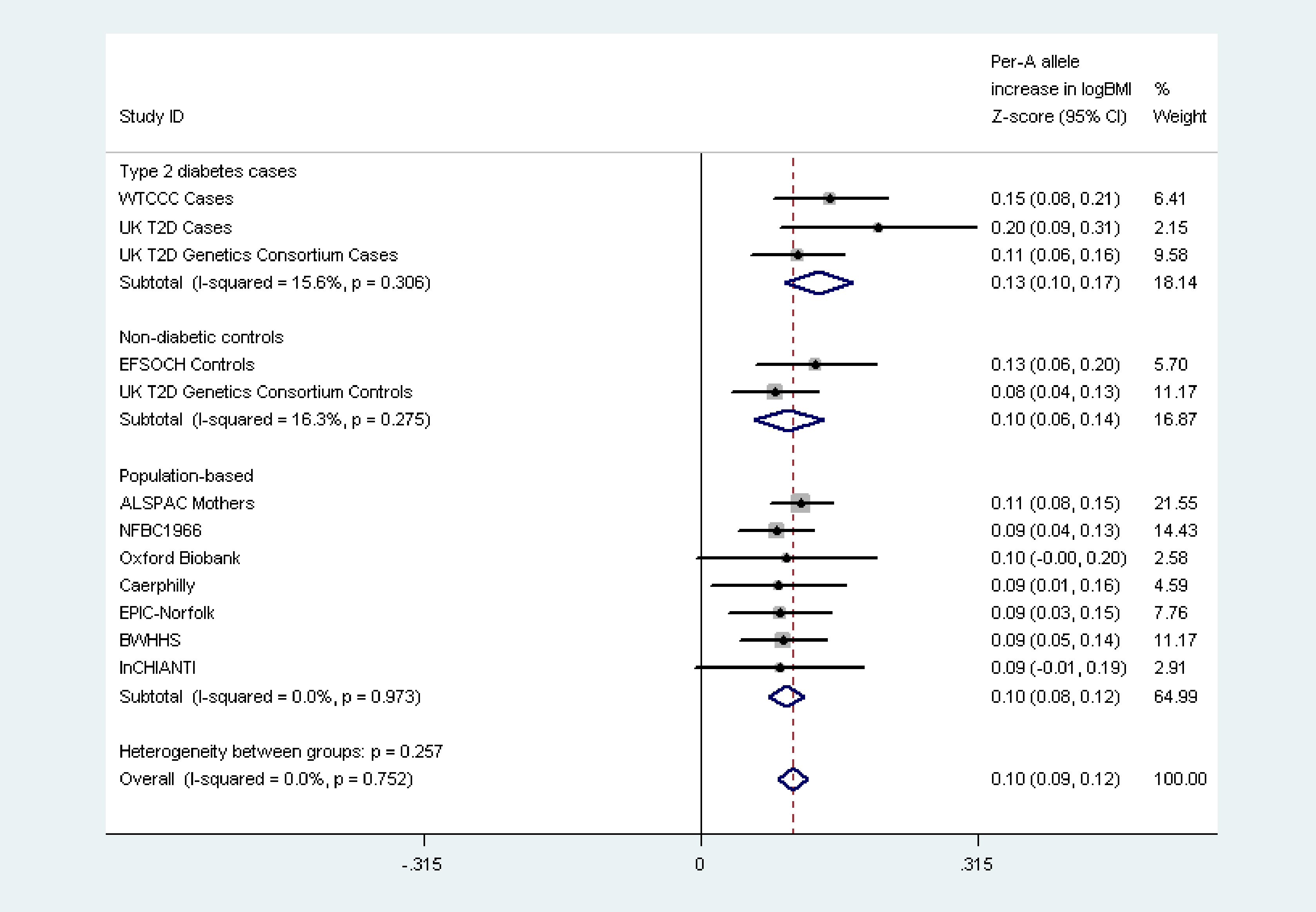

(a) Meta-analysis plot (adult cohorts) showing the rs9939609 per-A allele effect size on BMI, expressed in $\log_{10}$BMI Z-score units

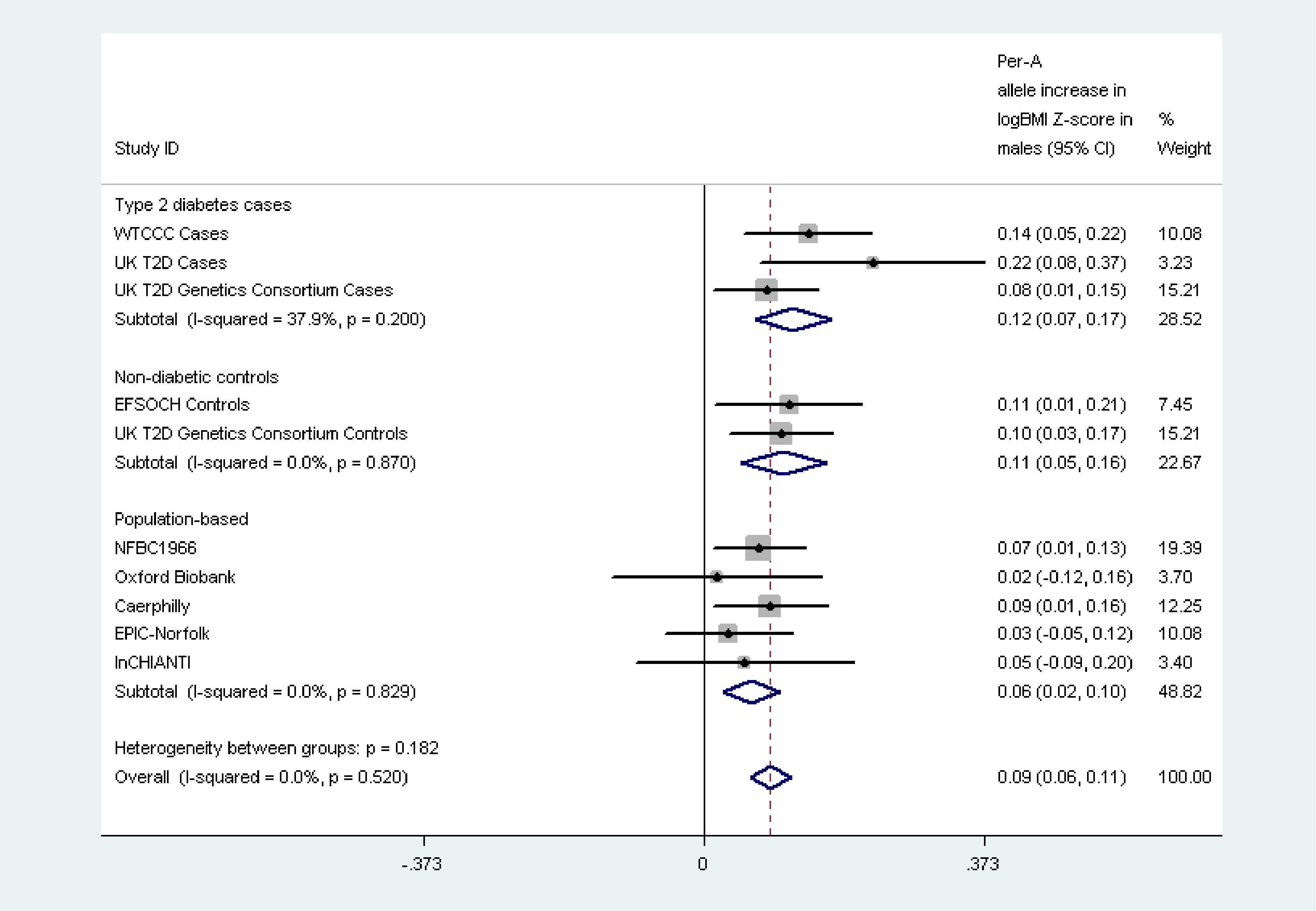

(b) Meta-analysis plot (adult cohorts) showing the rs9939609 per-A allele effect size on BMI in males only, expressed in log_{10}BMI Z-score units

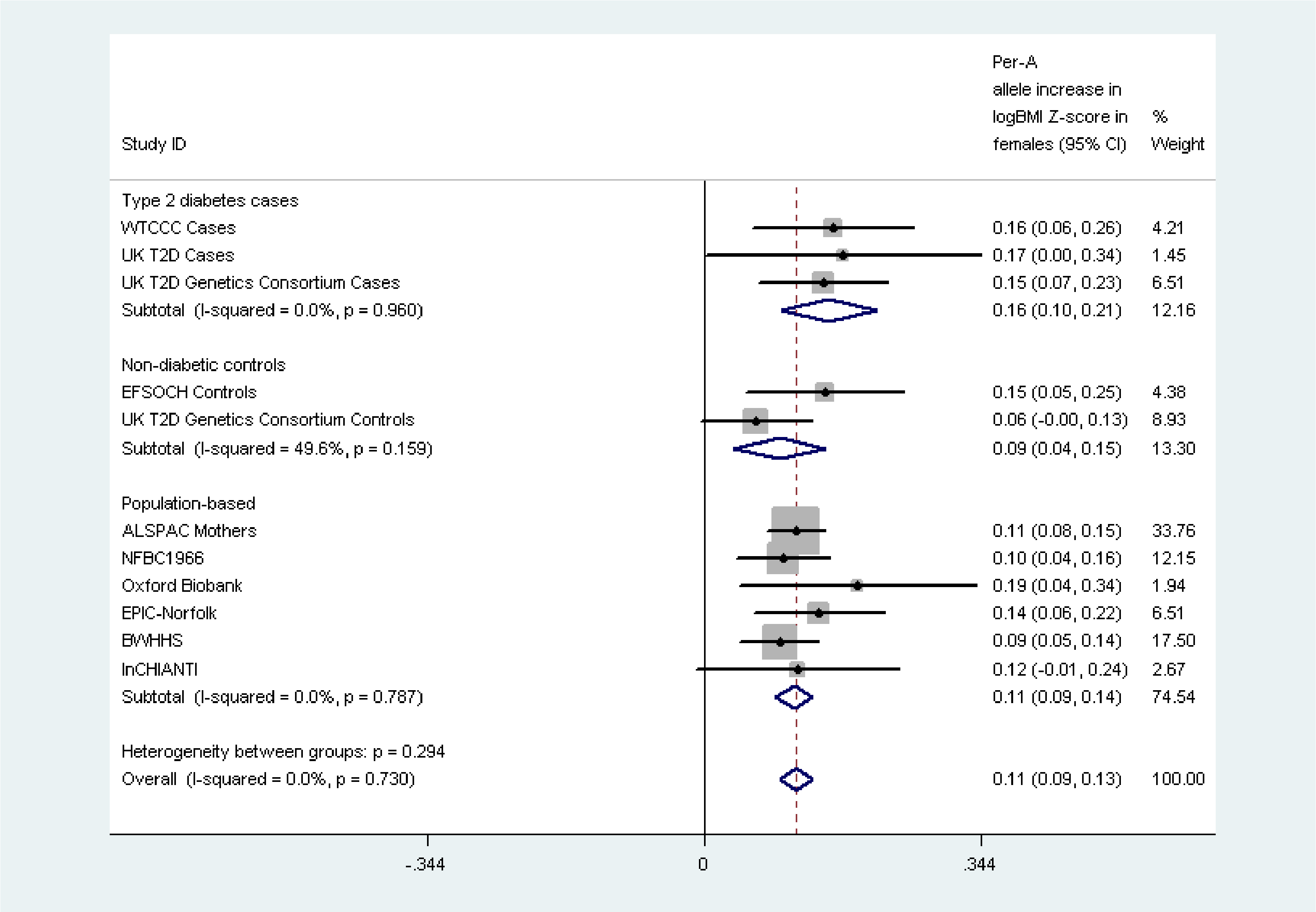

(c) Meta-analysis plot (adult cohorts) showing the rs9939609 per-A allele effect size on BMI in females only, expressed in $\log_{10}$BMI Z-score units

Figure S2

T2D
WTCCC
only

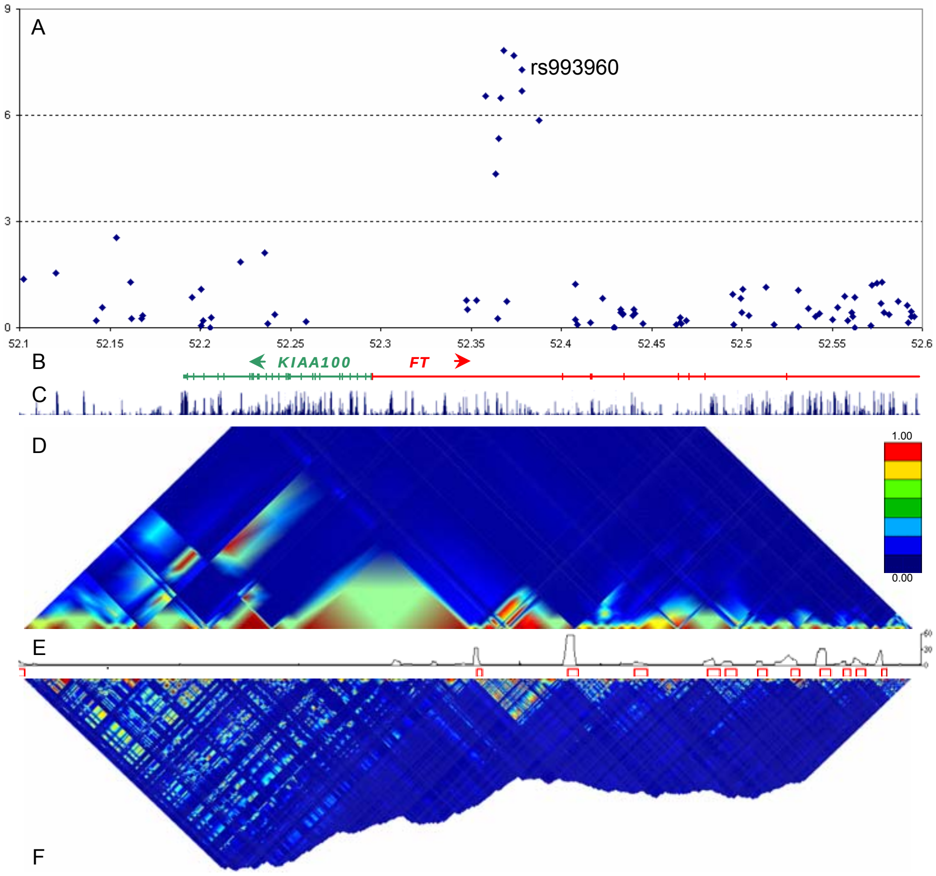

Association, gene structure, conservation, linkage disequilibrium and recombination for the *FTO* gene region. (a) T2D association in initial WTCCC study. A. Plot of -log(p-values) (Y-axis) for T2D against chromosome position in Mb(X-axis); B. Genomic location of genes showing intron and exon structure (NCBI BUILD 35); C. Multiz vertebrate alignment of 17 species showing evolutionary conservation; D. GOLDsurfer plot of linkage disequilibrium in CCC cases. Values given as pairwise r^2; E. Recombination rate given as cM/MB. Red lines represent recombination hotspots (HapMap); F. GOLDsurfer plot of linkage disequilibrium in HapMap CEU samples, values given as pairwise r^2.

BMI

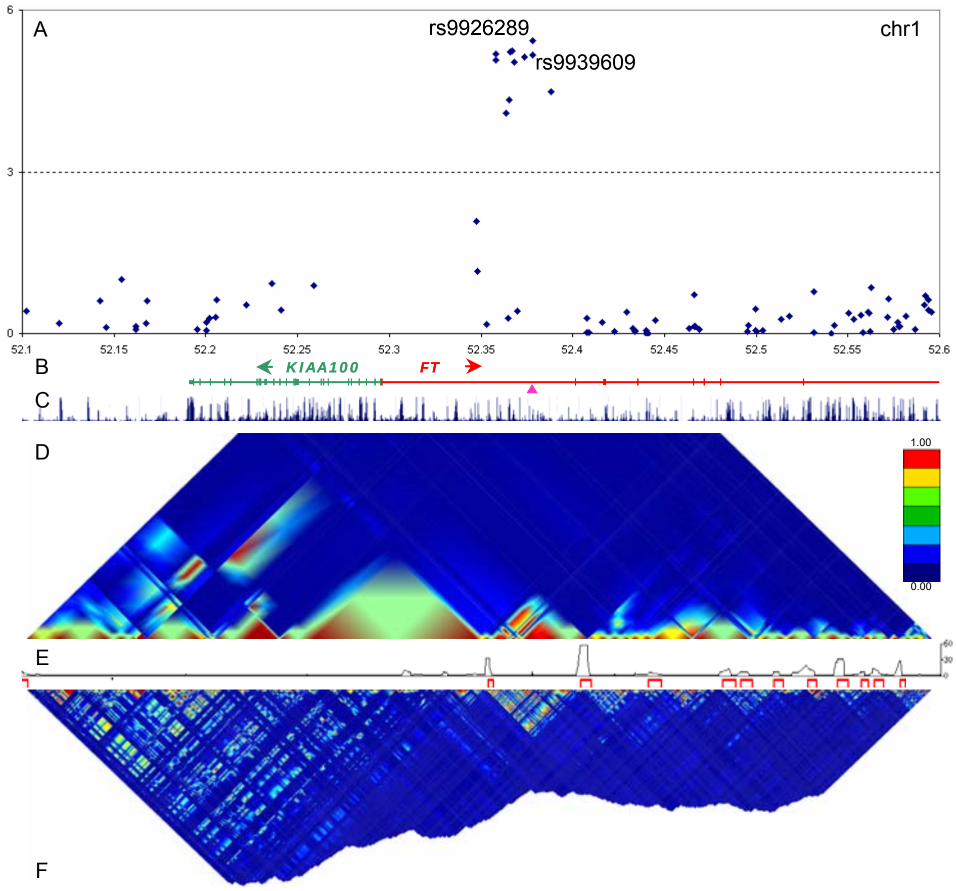

(b) BMI association in initial WTCCC study. A. Plot of -log(p-values) (Y-axis) for T2D against chromosome position in Mb(X-axis); B. Genomic location of genes showing intron and exon structure (NCBI BUILD 35); C. Multiz vertebrate alignment of 17 species showing evolutionary conservation; D. GOLDsurfer plot of linkage disequilibrium in CCC cases. Values given as pairwise r^2; E. Recombination rate given as cM/MB. Red lines represent recombination hotspots (HapMap); F. GOLDsurfer plot of linkage disequilibrium in HapMap CEU samples, values given as pairwise r^2.

Figure S3

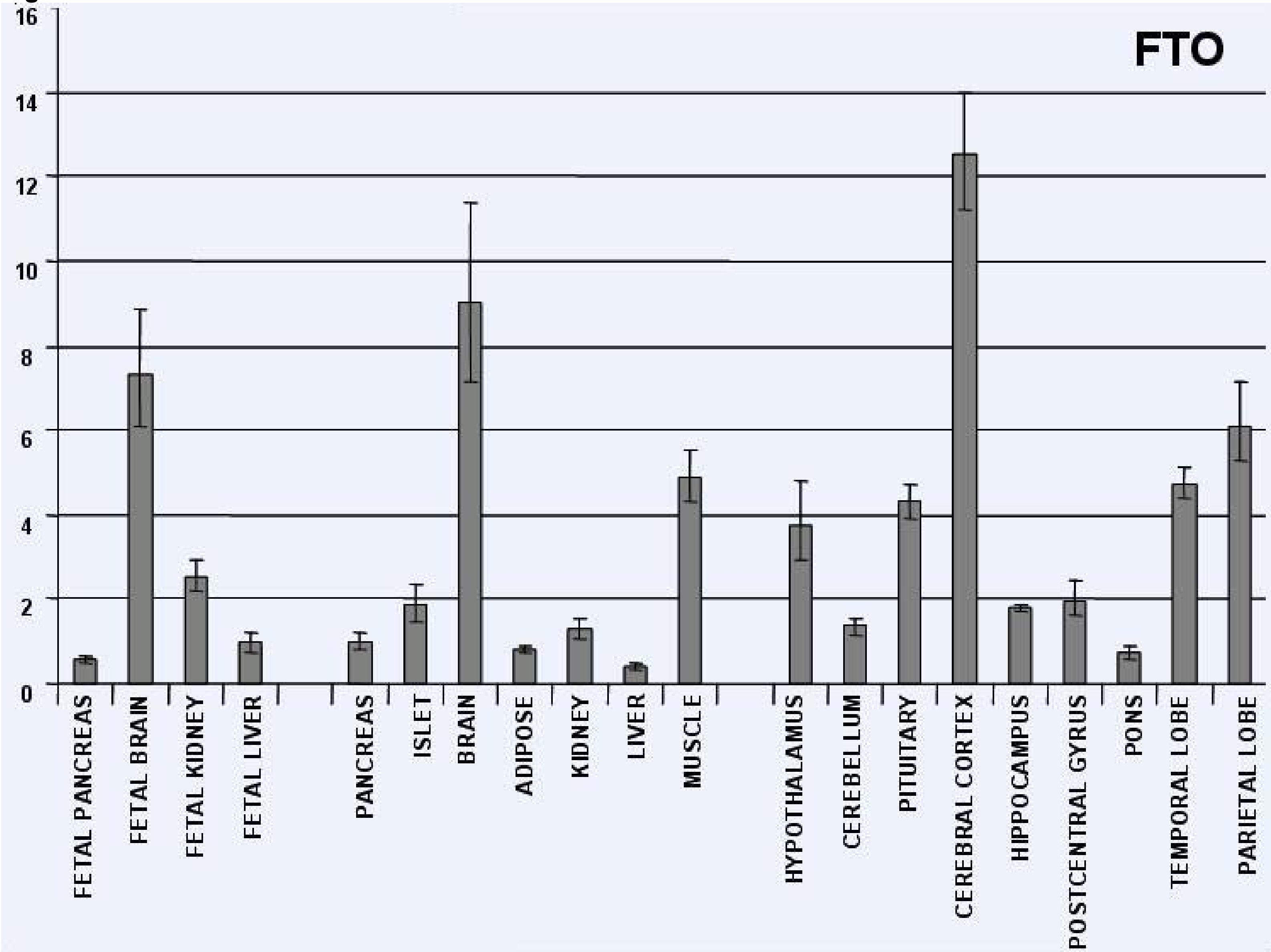

(a) Expression profile of the *FTO* gene. The relative expression level of the *FTO* gene is given for a range of human tissues. Figures on the Y-axis refer to the abundance of *FTO* mRNA relative to B2M and BGUS and are normalised to adult human pancreas.

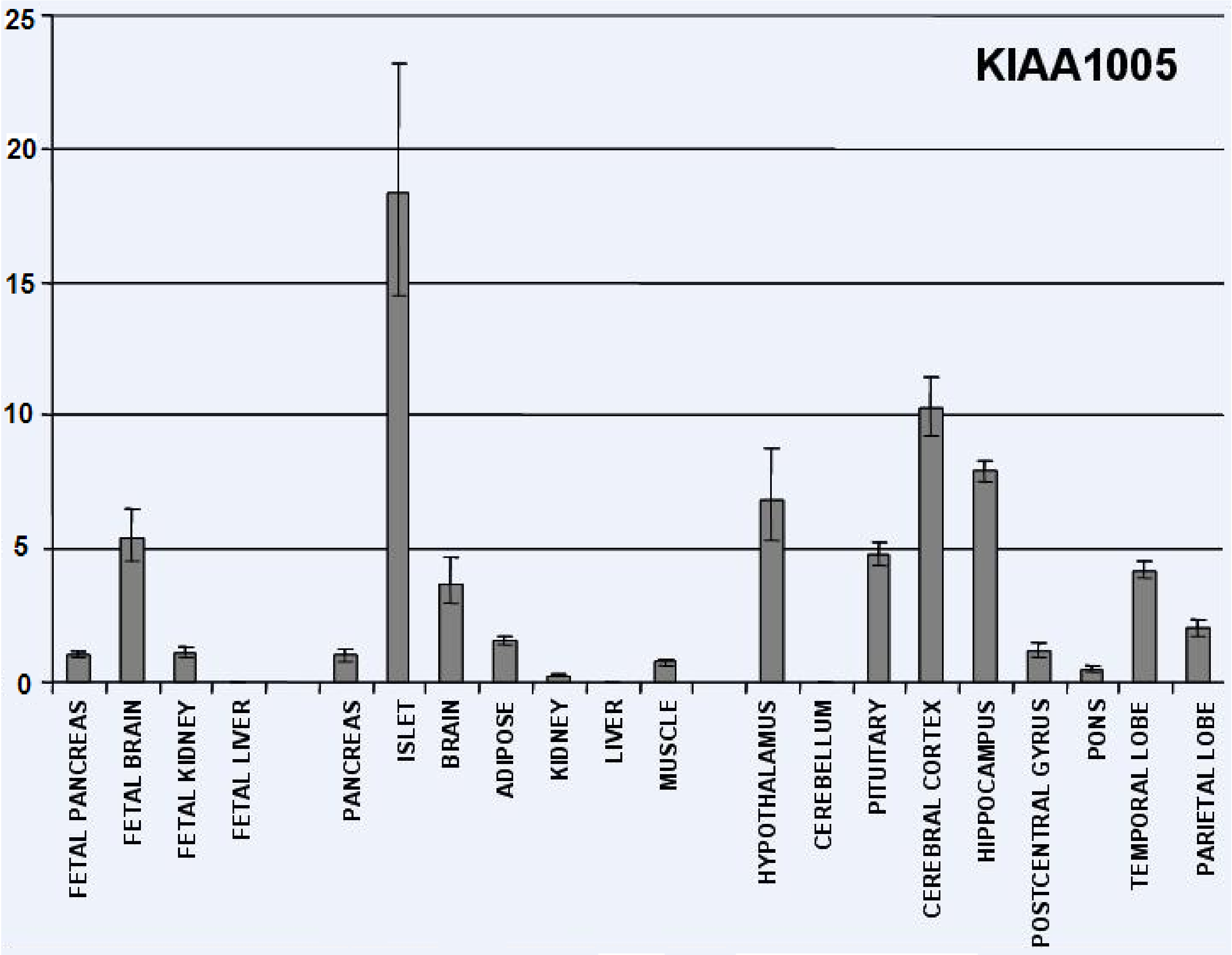

(b) Expression profile of *KIAA1005.* The relative expression level of the *KIAA1005* gene is given for a range of human tissues. Figures on the Y-axis refer to the abundance of *KIAA1005* mRNA relative to B2M and BGUS and are normalised to adult human pancreas.

Supporting tables

Table S1. Basic clinical characteristics of all studies

		N	%male	Age at study (yrs; mean, SD) or gestation (wks; mean, SD)	Average BMI (kg/m^2; geometric mean, SD range) or birth weight (g; mean, SD)
T2D Cases	UK cases (WTCCC)	1913	58	58.6 (10.3)	30.67 (25.37-37.08)
	UK T2D Cases	609	58	59.2(8.6)	31.49 (26.13-37.95)
	UKT2D Genetics Consortium Collection Cases	2961	57	64.1 (9.6)	31.02 (25.73, 37.38)
Controls	EFS	1746	51	31.8(5.6)	24.98 (21.17-29.47)
	UKT2D Genetics Consortium Collection Controls	3428	52	58.8 (11.9)	26.42 (26.35, 26.49)
Population participants					
Adult	ALSPAC	6376	0	28.4 (4.7)	22.70 (19.48, 26.46)
	NFBC1966	4435	48	31	24.37 (20.75, 28.63)
	Oxford Biobank	765	55	40.6 (6.1)	25.57 (21.85, 29.93)
Older adult					
	Caerphilly	1328	100	56.7 (4.5)	26.36 (23.01, 30.19)
	EPIC-Norfolk‡	2425	47	59.7 (9.0)	26.12 (22.64, 30.14)
	BWHHS	3244	0	68.8 (5.5)	27.15 (22.86, 32.26)
	InCHIANTI	851	45	74.3 (6.9)	27.15 (23.47, 31.42)
Obesity cases	EPIC-Norfolk obesity cases‡	1588	42	60.1 (8.8)	32.79 (30.22, 35.59)
Children*	ALSPAC	5010	49	11	18.76 (15.89, 22.14)
	NFBC1966	4203	47	14	19.23 (16.9, 21.87)
Birth†	ALSPAC	7477	51	39.7 (1.4)	3474 (479)
	NFBC1966	4320	47	40.2 (1.6)	3532 (503)

*ALSPAC children are offspring of the participants included in the adult study and data are given for eldest age (11 years) available. NFBC1966 children are the same participants as those in the adult study.
†ALSPAC birth data are for the same participants as those in the children study. NFBC1966 birth data are for the same participants as those in the children and adult studies. Twins were excluded from birth weight analyses in addition to those born before gestation of 36.00 weeks.
‡Of the 1588 EPIC-Norfolk obesity cases, 387 are also part of the EPIC-Norfolk population-based control-cohort.

Table S2(a). Genotype counts in normal weight and overweight (BMI ≥25kg/m^2) individuals from all studies – children and adults. Odds ratios and P values are corrected for sex.

Cohort	Normal weight				Overweight				Odds ratio (95% CI)	P
	**Total**	**TT**	**AT**	**AA**	**Total**	**TT**	**AT**	**AA**		
Type 2 diabetes										
**UK cases (WTCCC)**	257	87	142	28	1656	468	842	346	1.39 (1.15, 1.69)	0.001
**UK T2D Cases**	59	20	33	6	550	174	266	110	1.29 (0.87, 1.90)	0.21
**UKT2D GCC Cases**	324	113	174	37	2637	853	1306	478	1.22 (1.03, 1.44)	0.02
Combined T2D (I^2)									1.29 (1.14, 1.46)	7×10^{-5} (0%)
Controls										
**EFSOCH**	908	358	435	115	838	277	432	129	1.24 (1.06, 1.44)	0.006
**UKT2D Genetics Consortium Collection Controls**	1241	456	619	166	2187	798	1025	364	1.08 (0.98, 1.20)	0.14
Population participants										
Adult										
**ALSPAC**	5027	1879	2407	741	1349	419	676	254	1.24 (1.14, 1.36)	9×10^{-7}
**NFBC1966**	2657	1060	1211	386	1778	618	857	303	1.18 (1.08, 1.29)	0.0002
**Oxford Biobank**	361	138	175	48	404	149	181	74	1.15 (0.93, 1.41)	0.19
Older Adults										
**Caerphilly**	423	189	182	52	905	326	442	137	1.28 (1.08, 1.52)	0.004
**EPIC-Norfolk control-cohort**	923	351	443	129	1502	531	746	225	1.08 (0.95, 1.22)	0.24
**BWHHS**	1035	421	471	143	2209	785	1069	355	1.17 (1.05, 1.30)	0.004
**InCHIANTI**	249	82	127	40	602	187	289	126	1.15 (0.93, 1.41)	0.21
Combined population and control participants (I^2)									1.17 (1.12, 1.22)	4×10^{-14} (0%)
Children*										
**ALSPAC**										
**Aged 7**	5007	1861	2400	746	962	301	470	191	1.25 (1.13, 1.38)	9×10^{-6}
**Aged 8**	3736	1419	1761	556	1135	356	566	213	1.24 (1.13, 1.37)	8×10^{-6}
**Aged 9**	4130	1573	1942	615	1329	414	649	266	1.28 (1.17, 1.40)	4×10^{-8}
**Aged 10**	4022	1548	1878	596	1251	390	615	246	1.28 (1.17, 1.40)	7×10^{-8}
**Aged 11**	3706	1414	1740	552	1304	401	653	250	1.27 (1.16, 1.39)	2×10^{-7}
**NFBC1966**	3895	1490	1807	598	308	105	147	56	1.15 (0.98, 1.36)	0.09

*ALSPAC children are offspring of the participants included in the adult study and data are shown at 5 available ages. NFBC1966 children are the same participants as those in the adult study with data available at two time-points. Overweight is defined as 17.9, 18.4, 19.1, 19.8, 20.6 and 22.6 in boys aged 7, 8, 9, 10, 11 and 14, respectively, and as 17.8, 18.4, 19.1, 19.9, 20.7 and 23.3 in girls aged 7, 8, 9, 10, 11 and 14, respectively.

Table S2(b). Genotype counts in normal weight and obese (BMI ≥30kg/m^2) individuals from all studies – children and adults. Odds ratios and P values are corrected for sex.

Cohort	Normal weight				Obese				Odds ratio (95% CI)	
	**Total**	**TT**	**AT**	**AA**	**Total**	**TT**	**AT**	**AA**		P
Type 2 diabetes										
**UK cases (WTCCC)**	257	87	142	28	1011	257	516	238	1.58(1.29, 1.94)	1×10^{-5}
**UK T2D Cases**	59	20	33	6	357	110	167	80	1.38 (0.92, 2.06)	0.12
**UKT2D GCC cases**	324	113	174	37	1663	524	818	321	1.27 (1.07, 1.51)	0.008
Combined T2D (I^2)									1.39 (1.23, 1.57)	7×10^{-7} (22.2%)
Controls										
**EFSOCH**	908	358	435	115	242	67	138	37	1.41 (1.14, 1.76)	0.002
**UKT2D GCC Controls**	1241	456	619	166	659	214	309	136	1.28 (1.11, 1.46)	0.001
Population studies										
Adult										
**ALSPAC**	5027	1879	2407	741	353	107	157	89	1.43 (1.23, 1.67)	5×10^{-6}
**NFBC1966**	2657	1060	1211	386	415	132	193	90	1.36 (1.17, 1.57)	4×10^{-5}
**Oxford Biobank**	361	138	175	48	126	45	56	25	1.21 (0.90, 1.62)	0.20
Older Adult										
**Caerphilly**	423	189	182	52	200	67	101	32	1.37(1.07, 1.75)	0.01
**EPIC-Norfolk**†	923	351	443	129	1588	485	847	256	1.24 (1.09, 1.40)	0.0007
**BWHHS**	1035	421	471	143	840	274	427	139	1.26 (1.10, 1.44)	0.0007
**InCHIANTI**	249	82	127	40	212	66	92	54	1.26 (0.98, 1.64)	0.08
Combined population & control (I^2)									1.31(1.24, 1.38)	1×10^{-20} (0%)
Children*										
**ALSPAC**										
**Aged 7**	5007	1861	2400	746	251	82	121	48	1.20 (1.00, 1.44)	0.05
**Aged 8**	3736	1419	1761	556	266	88	124	54	1.24 (1.04, 1.48)	0.02
**Aged 9**	4130	1573	1942	615	322	89	167	66	1.39 (1.18, 1.63)	7×10^{-5}
**Aged 10**	4022	1548	1878	596	290	82	151	57	1.36 (1.15, 1.61)	4×10^{-4}
**Aged 11**	3706	1414	1740	552	283	79	149	55	1.35 (1.14, 1.61)	6×10^{-4}
**NFBC1966 Aged 14**	3895	1490	1807	598	45	9	20	16	2.14 (1.41, 3.25)	0.0003

*ALSPAC children are offspring of the participants included in the adult study and data are shown at 5 available ages. NFBC1966 children are the same participants as those in the adult study with data available at two time-points. Obesity is defined as 20.6, 21.6, 22.8, 24.0, 25.1 and 27.6 in boys aged 7, 8, 9, 10, 11 and 14, respectively, and as 20.5, 21.6, 22.8, 24.1, 25.4 and 28.6 in girls aged 7, 8, 9, 10, 11 and 14, respectively. †For this analysis, 1201 individuals from the EPIC-Norfolk obesity case group were added to supplement those in the EPIC-Norfolk population-based group with BMI of 30 kg/m^2 and above (N=387).

Table S3(a). Ponderal Index, corrected for sex and gestation in individuals born at 36.00 weeks' gestation or later (twins excluded)

Study	Total N	Mean ponderal index in kg/m^3 (95% CI) by child's genotype, corrected for sex and gestation			P value
		**TT**	**AT**	**AA**	
**ALSPAC**	5860	26.4 (26.3, 26.5)	26.4 (26.3, 26.5)	26.3 (26.1, 26.5)	0.29
**NFBC1966**	4292	27.4 (27.3, 27.5)	27.4 (27.3, 27.5)	27.6 (27.4, 27.8)	0.14

Table S3(b). The association of weight with rs9939609 genotypes, corrected for sex in a) type 2 diabetes cases from genome-wide and replication studies; b) control participants from genome-wide and replication studies and c) adult population studies. P values represent per-A allele effects.

Study	Total N	Mean weight in kg (95% CI) by genotype*			P
		**TT**	**AT**	**AA**	
a) Type 2 diabetes					
**UK cases (WTCCC)**	1913	86.6 (85.2, 88.1)	87.2 (86.1, 88.3)	91.9 (90.0, 93.8)	6×10^{-5}
**UK T2D Cases**	609	88.4 (86.1, 90.9)	88.9 (87.0, 90.9)	95.1 (91.8, 98.5)	0.005
**UKT2D Genetics Consortium Collection Cases**	2961	85.7 (84.6, 86.8)	86.6 (85.7, 87.5)	89.7 (88.1, 91.2)	1×10^{-4}
Combined T2D (I^2)					3×10^{-9} (0%)
b) Controls					
**EFSOCH**	1746	72.3 (71.3, 73.2)	73.9 (73.1, 74.7)	74.3 (72.8, 75.9)	0.007
**UKT2D Genetics Consortium Collection Controls**	3428	75.2 (74.5, 75.9)	75.7 (75.1, 76.3)	77.5 (76.3, 78.6)	0.002
c) Population participants					
Adult					
**ALSPAC**	6442	60.3 (60.1, 60.6)	60.9 (60.6, 61.3)	62.5 (61.8, 63.2)	7×10^{-8}
**NFBC1966**	4435	70.5 (69.9, 71.1)	71.5 (71.0, 72.1)	72.8 (71.8, 73.7)	2×10^{-5}
**Oxford Biobank**	765	74.0 (72.6, 75.5)	74.2 (72.9, 75.5)	77.2 (75.0, 79.5)	0.05
Older Adult					
**Caerphilly**	1328	76.2 (75.2, 77.2)	77.7 (76.8, 78.6)	78.3 (76.4, 80.3)	0.02
**EPIC-Norfolk**	2425	72.3 (71.6, 73.1)	73.3 (72.7, 74.0)	74.0 (72.9, 75.2)	0.008
**BWHHS**	3244	67.5 (66.8, 68.2)	68.6 (68.0, 69.3)	69.4 (68.3, 70.6)	0.002
**InCHIANTI**	856	67.6 (66.3, 68.9)	67.7 (66.6, 68.8)	69.1 (67.3, 70.8)	0.23
Combined population studies (I^2)					4×10^{-17} (0%)
Combined population and control studies (I^2)					7×10^{-21} (0%)
All studies (I^2)					2×10^{-28} (0%)

*Geometric mean and 95% confidence intervals are back-transformed from log_{10}(weight) values

Table S3(c). Children: weight and height corrected for sex

Study	Trait	Total N	Mean trait value (95% CI) by child's genotype			P value
			**TT**	**AT**	**AA**	
**ALSPAC**	**Weight aged 7 (kg)**	5970	25.3 (25.2, 25.5)	25.5 (25.3, 25.6)	25.9 (25.6, 26.1)	0.003
	**Weight aged 8 (kg)**	4983	29.4 (29.2, 29.7)	29.8 (29.6, 30.0)	30.5 (30.1, 30.9)	7×10^{-6}
	**Weight aged 9 (kg)**	5505	33.4 (33.1, 33.7)	34.1 (33.9, 34.4)	34.8 (34.3, 35.3)	1×10^{-7}
	**Weight aged 10 (kg)**	5309	36.5 (36.2, 36.8)	37.4 (37.1, 37.7)	38.2 (37.7, 38.8)	1×10^{-8}
	**Weight aged 11 (kg)**	5015	41.9 (41.5, 42.3)	42.7 (42.3, 43.1)	43.8 (43.2, 44.5)	4×10^{-7}
**NFBC1966**	**Weight aged 14 (kg)**	4255	50.7 (50.3, 51.1)	51.1 (50.7, 51.5)	51.5 (50.8, 52.2)	0.03
**ALSPAC**	**Height aged 7 (cm)**	5977	126.0 (125.7, 126.2)	125.9 (125.7, 126.1)	126.0 (125.7, 126.4)	0.90
	**Height aged 8 (cm)**	5154	132.5 (132.2, 132.8)	132.5 (132.2, 132.7)	132.8 (132.4, 133.2)	0.31
	**Height aged 9 (cm)**	5462	139.5 (139.3, 139.8)	139.7 (139.4, 139.9)	139.7 (139.3, 140.1)	0.50
	**Height aged 10 (cm)**	5285	143.9 (143.6, 144.2)	144.1 (143.8, 144.4)	144.4 (144.0, 144.8)	0.08
	**Height aged 11 (cm)**	5013	150.8 (150.4, 151.1)	150.8 (150.5, 151.1)	151.2 (150.7, 151.7)	0.20
**NFBC1966**	**Height aged 14 (cm)**	4393	162.9 (162.6, 163.3)	163.1 (162.8, 163.4)	163.1 (162.6, 163.7)	0.42

Means and 95% CIs for weight are back-transformed from logged values.

Table S3(d). The association of height with rs9939609 genotypes, corrected for sex in a) type 2 diabetes cases from genome-wide and replication studies; b) control participants from genome-wide and replication studies and c) adult population studies. P values represent per-A allele effects.

Study	Total N	Mean height in cm (95% CI) by genotype			P
		**TT**	**AT**	**AA**	
a) Type 2 diabetes					
**UK cases (WTCCC)**	1913	169.8 (169.2, 170.3)	169.5 (169.0, 169.9)	169.8 (169.1, 170.5)	0.87
**UK T2D Cases**	609	169.5 (168.6, 170.5)	169.3 (168.5, 170.1)	168.8 (167.6, 170.1)	0.39
**UKT2D Genetics Consortium Collection Cases**	2961	167.6 (167.2, 168.1)	167.5 (167.2, 167.8)	167.7 (167.1, 168.3)	0.89
b) Controls					
**EFSOCH**	1746	172.0 (171.5, 172.5)	171.4 (171.0, 171.9)	171.3 (170.5, 172.1)	0.06
**UKT2D Genetics Consortium Collection Controls**	3428	169.5 (169.2, 169.9)	169.8 (169.5, 170.2)	169.4 (168.8, 170.0)	0.89
c) Population participants					
Adult					
**ALSPAC**	6442	164.2 (163.9, 164.5)	163.9 (163.7, 164.1)	164.0 (163.6, 164.4)	0.23
**NFBC1966**	4588	171.1 (170.8, 171.4)	171.2 (170.9, 171.5)	171.3 (170.8, 171.8)	0.32
**Oxford Biobank**	765	170.7 (170.0, 171.5)	171.3 (170.6, 172.0)	171.2 (170.0, 172.3)	0.38
Older Adult					
**Caerphilly**	1328	171.0 (170.5, 171.6)	171.4 (170.9, 171.9)	171.4 (170.5, 172.4)	0.36
**EPIC-Norfolk**	2425	167.5 (167.0, 167.9)	167.5 (167.2, 167.9)	167.0 (166.3, 167.6)	0.37
**BWHHS**	3244	159.0 (158.7, 159.4)	158.6 (158.3, 158.9)	158.8 (158.3, 159.3)	0.24
**InCHIANTI**	851	158.5 (157.7, 159.4)	158.8 (158.2, 159.5)	157.9 (156.8, 159.0)	0.49

Table S3(e). The association of waist circumference with rs9939609 genotypes, corrected for sex in a) type 2 diabetes cases from genome-wide and replication studies; b) control participants from genome-wide and replication studies and c) adult population studies. P values represent per-A allele effects.

Study	Total N	Mean waist circumference in cm (95% CI) by genotype			P
		**TT**	**AT**	**AA**	
a) Type 2 diabetes					
**UK cases (WTCCC)**	1913	102.6 (101.5, 103.8)	103.2 (102.4, 104.1)	106.1 (104.6, 107.6)	0.001
**UK T2D Cases**	588	104.3 (102.5, 106.2)	104.6 (103.1, 106.1)	110.3 (107.7, 112.9)	0.001
**UKT2D Genetics Consortium Collection Cases**	2948	102.8 (102.0, 103.7)	103.6 (103.0, 104.3)	106.1 (104.9, 107.3)	3×10^{-5}
Combined T2D (I^2)					2×10^{-9} (0%)
b) Controls					
**EFSOCH***	893	90.2 (89.0, 91.3)	91.8 (90.9, 92.8)	93.1 (91.3, 95.0)	0.004
**UKT2D Genetics Consortium Collection Controls**	3425	91.0 (90.4, 91.6)	90.9 (90.4, 91.5)	92.8 (91.8, 93.7)	0.01
c) Population participants					
Adult					
**ALSPAC**	NA	NA	NA	NA	NA
**NFBC1966**	4425	82.3 (81.8, 82.8)	83.2 (82.8, 83.7)	84.1 (83.4, 85.0)	4×10^{-5}
**Oxford Biobank**	765	84.0 (82.8, 85.1)	84.5 (83.5, 85.6)	86.6 (84.7, 88.5)	0.029
Older Adult					
**Caerphilly**	NA	NA	NA	NA	NA
**EPIC-Norfolk**	2425	87.4 (86.7, 88.1)	88.2 (87.6, 88.8)	89.1 (88.0, 90.1)	0.007
**BWHHS**	3225	84.6 (83.9, 85.3)	85.7 (85.2, 86.4)	86.0 (84.9, 87.0)	0.01
**InCHIANTI**	856	91.5 (90.3, 92.7)	92.0 (91.0, 92.9)	93.2 (91.7, 94.8)	0.09
Combined population studies (I^2)					4×10^{-9} (0%)
Combined population and control studies (I^2)					3×10^{-11} (0%)
All studies (I^2)					9×10^{-19} (0%)

NA, not available. All means and 95% CIs are backtransformed from logged values. *Available for males only, since females were pregnant at time of study.

Table S3(f). The association of various measures of subcutaneous fat with rs9939609 genotypes, corrected for sex in four studies. P values represent per-A allele effects.

Trait and study	Total N	Mean trait value (95% CI) by genotype TT	AT	AA	P
**a) Biceps skinfold thickness (mm)**					
EFSOCH	1745	8.0 (7.8, 8.3)	8.4 (8.2, 8.7)	8.8 (8.4, 9.3)	0.002
Oxford Biobank	761	9.0 (8.5, 9.6)	9.2 (8.8, 9.7)	9.7 (8.9, 10.6)	0.18
Caerphilly	1113	6.1 (5.9, 6.4)	6.3 (6.1, 6.5)	6.6 (6.1, 7.0)	0.05
Combined biceps (I^2)	3619	Per-A allele Z score increase (95% CI): 0.10 (0.05, 0.14)			0.0002 (0%)
**b) Triceps skinfold thickness (mm)**					
EFSOCH	1746	14.0 (13.6, 14.4)	14.3 (13.9, 14.7)	14.9 (14.2, 15.6)	0.04
Oxford Biobank	761	17.2 (16.3, 18.0)	17.3 (16.6, 18.1)	18.7 (17.3, 20.1)	0.11
Caerphilly	1110	10.5 (10.2, 10.9)	10.7 (10.4, 11.0)	10.9 (10.2, 11.5)	0.33
InCHIANTI	863	15.2 (14.4, 16.1)	15.3 (14.6, 16.0)	17.3 (16.1, 18.6)	0.01
Combined triceps (I^2)	4480	Per-A allele Z score increase (95% CI): 0.08 (0.03, 0.12)			0.0009 (0%)
**c) Subscapular skinfold thickness (mm)**					
EFSOCH	1743	17.4 (16.8, 17.9)	18.3 (17.8, 18.8)	19.2 (18.3, 20.2)	0.0005
Oxford Biobank	763	17.9 (17.0, 18.9)	18.7 (17.8, 19.6)	19.5 (18.0, 21.2)	0.07
Caerphilly	1108	16.0 (15.5, 16.6)	16.6 (16.1, 17.1)	17.3 (16.3, 18.3)	0.02
Combined subscap (I^2)	3608	Per-A allele Z score increase (95% CI): 0.11 (0.06, 0.16)			1×10^{-5} (0%)
**d) Sum of skinfolds (biceps, triceps & subscap; mm)**					
EFSOCH	1740	52.8 (51.3, 54.3)	55.1 (53.7, 56.4)	57.8 (55.2, 60.6)	0.001
Oxford Biobank	760	45.5 (43.4, 47.7)	46.7 (44.8, 48.7)	49.7 (46.3, 53.3)	0.05
Caerphilly	1102	33.1 (32.1, 34.1)	34.1 (33.2, 35.0)	35.1 (33.2, 37.1)	0.04
Combined sumskin (I^2)	3602	Per-A allele Z score increase (95% CI): 0.11 (0.06, 0.16)			2×10^{-5} (0%)
**e) Suprailiac skinfold thickness (mm)**					
Oxford Biobank	765	17.8 (16.7, 19.0)	18.4 (17.4, 19.5)	19.4 (17.6, 21.4)	0.02
**f) Mid-arm circumference (cm)**					
EFSOCH	1744	30.1 (29.8, 30.3)	30.6 (30.3, 30.8)	30.6 (30.2, 31.0)	0.005
InCHIANTI	883	28.2 (27.8, 28.6)	28.6 (28.3, 28.9)	29.4 (29.0, 30.0)	0.0002
Combined mid-arm circumference (I^2)	2627	Per-A allele Z score increase (95% CI): 0.13 (0.07, 0.18)			2×10^{-5} (43.5%)

With the exception of mid-arm circumference, geometric means and 95% confidence intervals are presented, back-transformed from logged values

Table S4. Sequencing variants of *FTO* gene, observed in 47 individuals with BMI >40 kg/m^2.

FTO region	Amino Acid change	Nucleotide change†	Chromosome 16 coordinate‡ (UCSC)	rs number	Number observed	
					Hom	Het
Exon3	N186N	558C>CT	52417711	-		1
3’UTR	-	1518+363del A	52703691	-		1
3’UTR	-	1518+888G>A	57704216	-		2
3’UTR	-	1518+1379delG	52704708	5816925	29	11
3’UTR	-	1518+2105G>A	52705436	708277	27	9

The r^2 value between rs9939609 and the two common variants is 0 and they therefore cannot explain the observed association.
Amino acid change for variants identified in non-protein coding regions is not applicable and denoted by -. †Nucleotide change numbering based on coding nucleotides where A of translation start-codon is number 1. ‡Chromosome 16 coordinates from UCSC Genome Browser (http://genome.ucsc.edu) using the human assembly (Mar. 2006).

Supporting references

S1. G. W. Mills *et al.*, *Diabetologia* 47, 732 (Apr, 2004).

S2. K. R. Owen, A. Stride, S. Ellard, A. T. Hattersley, *Diabetes Care* 26, 2088 (Jul, 2003).

S3. B. Knight, B. M. Shields, A. T. Hattersley, *Paediatr Perinat Epidemiol* 20, 172 (Mar, 2006).

S4. J. Golding, M. Pembrey, R. Jones, *Paediatr Perinat Epidemiol* 15, 74 (Jan, 2001).

S5. R. W. Jones *et al.*, *Eur J Hum Genet* 8, 653 (Sep, 2000).

S6. I. S. Rogers *et al.*, *Am J Clin Nutr* 84, 739 (Oct, 2006).

S7. S. D. Leary *et al.*, *Obesity (Silver Spring)* 14, 2284 (Dec, 2006).

S8. P. Rantakallio, *Paediatr Perinat Epidemiol* 2, 59 (Jan, 1988).

S9. M. R. Jarvelin *et al.*, *Hypertension* 44, 838 (Dec, 2004).

S10. G. D. Tan *et al.*, *Diabetologia* 49, 158 (Jan, 2006).

S11. *J Epidemiol Community Health* 38, 259 (Sep, 1984).

S12. A. M. Fehily, B. K. Butland, J. W. Yarnell, *Eur J Clin Nutr* 44, 107 (Feb, 1990).

S13. N. Day *et al.*, *Br J Cancer* 80 Suppl 1, 95 (Jul, 1999).

S14. D. A. Lawlor, C. Bedford, M. Taylor, S. Ebrahim, *J Epidemiol Community Health* 57, 134 (Feb, 2003).

S15. L. Ferrucci *et al.*, *J Am Geriatr Soc* 48, 1618 (Dec, 2000).

S16. B. Bartali *et al.*, *Soz Praventivmed* 47, 336 (2002).

S17. T. J. Cole, M. C. Bellizzi, K. M. Flegal, W. H. Dietz, *Bmj* 320, 1240 (May 6, 2000).

S18. J. P. Higgins, S. G. Thompson, J. J. Deeks, D. G. Altman, *Bmj* 327, 557 (Sep 6, 2003).
